# Prevalence of drug-resistant pulmonary tuberculosis in India: systematic review and meta-analysis

**DOI:** 10.1186/s12889-017-4779-5

**Published:** 2017-10-17

**Authors:** Vishal Goyal, Vijay Kadam, Prashant Narang, Vikram Singh

**Affiliations:** Medical Affairs, Janssen India, Johnson & Johnson Pvt Ltd., Arena Space, 8th floor, Off JVLR, Jogeshwari (E), Mumbai, 400060 India

**Keywords:** Drug-resistant tuberculosis, India, Prevalence

## Abstract

**Background:**

Drug-resistant pulmonary tuberculosis (DR-TB) is a significant public health issue that considerably deters the ongoing TB control efforts in India. The purpose of this review was to investigate the prevalence of DR-TB and understand the regional variation in resistance pattern across India from 1995 to 2015, based on a large body of published epidemiological studies.

**Methods:**

A systematic review of published studies reporting prevalence of DR-TB from biomedical databases (PubMed and IndMed) was conducted. Meta-analysis was performed using random effects model and the pooled prevalence estimate (95% confidence interval [CI]) of DR-TB, multidrug resistant (MDR-) TB, pre-extensively drug-resistant (pre-XDR) TB and XDR-TB were calculated across two study periods (decade 1: 1995 to 2005; decade 2: 2006 to 2015), countrywide and in different regions. Heterogeneity in this meta-analysis was assessed using I^2^ statistic.

**Results:**

A total of 75 of 635 screened studies that fulfilled the inclusion criteria were selected. Over 40% of 45,076 isolates suspected for resistance to any first-line anti-TB drugs tested positive. Comparative analysis revealed a worsening trend in DR-TB between the two study decades (decade 1: 37.7% [95% CI = 29.0; 46.4], *n* = 25 vs decade 2: 46.1% [95% CI = 39.0; 53.2], *n* = 36). The pooled estimate of MDR-TB resistance was higher in previously treated patients (decade 1: 29.8% [95% CI = 20.7; 39.0], *n* = 13; decade 2: 35.8% [95% CI = 29.2; 42.4], *n* = 24) as compared with the newly diagnosed cases (decade 1: 4.1% [95% CI = 2.7; 5.6], n = 13; decade 2: 5.6% [95% CI = 3.8; 7.4], *n* = 17). Overall, studies from Western states of India reported highest prevalence of DR-TB (57.8% [95% CI = 37.4; 78.2], *n* = 6) and MDR-TB (39.9% [95% CI = 21.7; 58.0], n = 6) during decade 2. Prevalence of pre-XDR TB was 7.9% (95% CI = 4.4; 11.4, *n* = 5) with resistance to fluoroquinolone (66.3% [95% CI = 58.2; 74.4], n = 5) being the highest. The prevalence of XDR-TB was 1.9% (95% CI = 1.2; 2.6, *n* = 14) over the 20-year period.

**Conclusion:**

The alarming increase in the trend of anti-TB drug resistance in India warrants the need for a structured nationwide surveillance to assist the National TB Control Program in strengthening treatment strategies for improved outcomes.

## Background

Accelerated tuberculosis (TB) control efforts have been threatened by the emergence of *Mycobacterium tuberculosis* strains that are resistant to potent first-line drugs (drug resistant tuberculosis or DR-TB) [1–3]. In 2015, the World Health Organization (WHO) estimated 480,000 incident multidrug resistant TB (MDR-TB; resistance of both isoniazid and rifampicin) cases globally. With an estimated 79,000 MDR-TB cases, India along with the Russian Federation and South Africa accounted for 45% of the total notified combined MDR-TB and rifampicin-resistant (RR-TB) cases in 2015 [4].

The management of DR-TB is critical and based on laboratory confirmation of TB and a clear understanding of drug resistance aided by drug susceptibility testing (DST) to ensure accurate diagnosis and early intervention of appropriate treatment [1, 3, 5]. Currently, the WHO recommended treatment strategy for complex MDR-TB comprises of a minimum of 5 drugs (including an injectable aminoglycoside) and a protracted treatment period of 18 to 24 months [1, 2]. However, only 50% of patients worldwide with MDR-TB achieve successful completion of treatment, partially owing to high death rates (250,000 [range, 16,000–340,000] estimated deaths from MDR-TB/RR-TB in 2015) and loss to follow-up [2, 4, 6]. In India, only 46% patients with MDR-TB have been reported to achieve treatment success in 2015 (vs 48% patients who achieved treatment success in 2014) with 20% each of death and lost to follow-up [7, 8]. Further, worsening outcome of extensively drug-resistant TB (XDR-TB; resistance to at least one fluoroquinolone and injectable aminoglycoside in addition to MDR-TB) has been reported in 9.5% patients with MDR-TB in 2015 [4].

Prevention and control of drug resistance is therefore strongly recommended by the WHO through implementation of routine surveillance systems driven by systematic DST [3, 9, 10]. Nationwide survey conducted in representative populations using standardized patient stratification and employing quality-assured rapid diagnostic methods are fundamental to a strengthened surveillance [9]. The Revised National Tuberculosis Control Programme (RNTCP) endorses the WHO recommended Directly Observed Treatment, Short course (DOTS) and systematic surveillance in India. This initiative was introduced in 1997 and achieved nationwide coverage in 2006 [8, 11]. Improvements in RNTCP surveillance approach have been noted in the recent years and India accounted for 27% of the global TB notifications in 2014 (12% from private sectors) [3, 12]. However, India remains one of the six countries with an enormous MDR-TB burden that failed to implement a nationwide drug-resistance surveillance (DRS) and relies largely on a sub-national evaluation approach [3, 8].

Currently, published studies have reported the prevalence of DR-TB from region-specific data obtained from city or state government health facilities or private set-ups. Epidemiological interpretations from these studies are challenged by large variations in research methodology, patient selection, diagnostic methods, unclear definitions of retreatment as well as data analysis and reporting. Further, till date, there has been no attempt to consolidate these studies to derive pooled prevalence estimates of DR-TB and stratify the prevalence based on geographical distribution. The present study was therefore designed to provide pooled estimates for DR-TB (MDR-TB, pre-XDR and XDR-TB) in India through systematic review and meta-analysis of published studies conducted across two decades (1995 to 2015).

## Methods

### Search strategy

Published studies of DR-TB in India were searched using the National Library of Medicine’s database, PubMed. Free text and index terms (Medical Subject Headings) related to DR-TB, India and prevalence were used and a wide search strategy was employed to maximize retrieval of relevant articles. Using elements of PICO, the following search terms were identified, Population: patients from India (*India*); Outcome: prevalence of drug resistant tuberculosis (*prevalence, incidence, epidemiology, tuberculosis, Mycobacterium tuberculosis, drug resistant tuberculosis, multidrug resistant tuberculosis, MDR-TB, extensively drug resistant tuberculosis, XDR-TB, anti-tuberculosis drug resistance, totally drug-resistant tuberculosis, TDR-TB*). Published articles indexed only in the Indian database IndMed (http://indmed.nic.in/) and not in PubMed were retrieved using similar search terms. To maximize search results, bibliographies of other reviews and original studies were searched manually for additional relevant studies.

### Definitions, data extraction, and analysis

The term drug resistance or DR-TB was used for mono-drug resistance (resistance to one first-line anti-tubercular drug only) and poly-drug resistance (resistance to more than one first-line anti-TB drug other than both isoniazid and rifampicin). Multidrug resistance or MDR-TB was defined as TB with resistance to at least both isoniazid and rifampicin. Pre–XDR was referred to as multidrug resistance along with resistance to a fluoroquinolone or second-line injectable agent but not both. Finally, resistance to any fluoroquinolone and at least one of three second-line injectable drugs (capreomycin, kanamycin and amikacin), in addition to multidrug resistance was referred to as extensively drug resistance or XDR-TB. Previously treated patients included those receiving ≥1 month of anti-TB drugs in the past and newly diagnosed patients were those who were never treated for TB or had taken anti-TB drugs for less than 1 month.

The list of articles with studies conducted within decades 1995 to 2005 and 2006 to 2015 retrieved from the two databases were screened and selected manually based on title and abstract to identify relevant studies for inclusion. Once the initial overview was completed, critical literature appraisal of the relevant articles based on the abstract or full-text was performed by a specifically developed data evaluation spreadsheet. Key items included in the spreadsheet were: region of sample origin (including city or state), study period, prevalence of DR-TB (including MDR-TB, pre-XDR and XDR-TB), case-wise prevalence of DR-TB (newly diagnosed or previously treated or any other type as specified in individual studies), pattern of drug resistance (mono- and combined drug resistance), HIV status and diagnostic techniques used for detection of drug susceptibility (phenotypic or genotypic techniques). A substantial degree of variability in research methodology with respect to patient selection and calculation of prevalence of drug resistance was noted. Calculation of prevalence of DR, MDR (all cases, previously treated, new and combined), pre-XDR and XDR for individual studies were performed using the following standard formulae to maintain uniformity and to assist interpretation.

$$ \%\mathrm{prevalence}\  \mathrm{of}\ \mathrm{DR}/\mathrm{MDR}/\mathrm{pre}\hbox{-} \mathrm{XDR}/\mathrm{XDR}\hbox{-} \mathrm{TB}=\frac{\mathrm{Number}\  \mathrm{of}\  \mathrm{cases}\ \left(\mathrm{DR}/\mathrm{MDR}/\mathrm{pre}\hbox{-} \mathrm{XDR}/\mathrm{XDR}\hbox{-} \mathrm{TB}\right)}{\mathrm{Total}\  \mathrm{number}\  \mathrm{of}\;M. tuberculosis\;\mathrm{isolates}\  \mathrm{available}\ \mathrm{for}\ \mathrm{drug}\  \mathrm{susceptibility}\  \mathrm{testing}}\times 100 $$

For prevalence of previously treated and newly diagnosed cases of MDR, pre-XDR and XDR-TB, the number of previously treated or newly diagnosed *M. tuberculosis* (MTB) isolates were considered.

The studies were stratified based on predefined variables to understand variations in prevalence estimates. The subgroup analysis was performed on the following variables: 1) By decade: decade 1 (1995 to 2005), period during the initial years of RNTCP implementation and decade 2 (2006 to 2015), period during which RNTCP achieved national coverage 2) By region: North India included states, Jammu and Kashmir, Himachal Pradesh, Punjab, Uttaranchal, Haryana, Delhi, Rajasthan, Uttar Pradesh, Bihar and Jharkhand; South India: Andhra Pradesh, Karnataka, Kerala and Tamil Nadu; West India: Gujarat, Maharashtra and Goa; East and central India: West Bengal, Orissa, all north-eastern states, Chhattisgarh and Madhya Pradesh.

### Eligibility

Studies were considered eligible for inclusion based on the following criteria: (1) specifically reporting the prevalence of pulmonary DR-TB, including breakdown by type of DR-TB (MDR-TB, pre-XDR or XDR-TB) in a population, subgroup or community exclusively from India (2) reporting detection of DR-TB by phenotypic or genotypic assays and suggestive of trends in resistance patterns for anti-TB drugs in isolates of MTB (3) conducted during the years 1995 to 2015.

Articles not published in English and not reporting epidemiology data on DR-TB were excluded. Additionally, the following studies were excluded: (1) reporting prevalence data on non-Indian populations or multicenter studies in which separation of Indian population’s DR-TB status was not possible (2) comparing or validating diagnostic tests for DR-TB detection and treatment outcomes or studies on gene mutation profiling with no epidemiological impact (3) reporting both pulmonary and extra pulmonary TB cases wherein isolation of pulmonary data was not possible (4) involving an exclusively human immunodeficiency virus (HIV) co-infected population. Case studies, editorials, author responses, commentaries and general reviews and expert opinions (to avoid duplication) were also excluded.

### Statistical analysis

Meta-analysis was undertaken using random effects model and the pooled estimate for the prevalence of drug resistance along with 95% CI were calculated. Subgroup analyses were used to understand the potential influences on prevalence estimates. Prevalence estimates were compared descriptively by decade, region and type of resistance (previously treated or newly diagnosed) [13]. Heterogeneity among studies was quantified using the I^2^ statistic. An I^2^ value of 0% indicates no observed heterogeneity whereas, higher values signify increasing heterogeneity. The negative values of I^2^ were set to zero in order to get all values between 0% and 100% [14]. All analyses were performed using SAS version 9.4.

## Results

### Summary of literature search

**Fig. 1:**
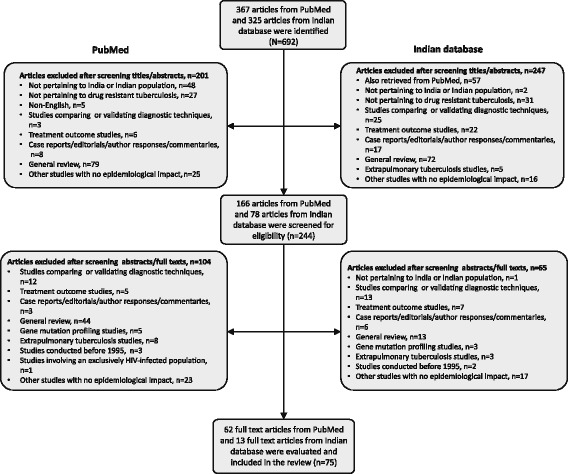
Flow diagram for selection of studies

The literature search identified a total of 635 articles (PubMed, *n* = 367; Indian database, *n* = 268) of which based on the inclusion and exclusion criteria, a total 75 articles from both databases (PubMed, *n* = 62; Indian database, *n* = 13) were included in this review (Fig. 1).

### Summary of key study characteristics

Characteristics of the 75 articles included are summarized in Table 1. North India had the largest number of studies (*n* = 32), followed by South India (*n* = 25), West India (*n* = 12), East India (*n* = 4) and Central India (*n* = 2). The results from East and Central regions were combined and populated together for the subgroup analysis, due to smaller number of studies.

**Table 1:** Characteristics of studies included in the review

S. No	Study (Citation)	City	Study Year	Patient population	MTB isolates total (n)	DR-TB^a^ (%)	Mono-drug resistance (%)					MDR-TB (%)			Pre-XDR^e^ (%)			XDR^f^ (%)	DST method	Ref population
							H	R	E	S	Z	Overall^b^	Previously treated^c^ (%)	Newly diagnosed cases^d^ (%)	Total	FQ	Inj			
NORTH INDIA																				
1	Malhotra B et al., 2002 [50]	Jaipur	1997–1999	Combined PTB patients	122	36.1	13.1	3.3				17.2	24.4	4.6					LJ	Patients attending medical college
2	Sharma S K et al.(a)., 2009 [51]	Delhi	1997–2003	Prev. treated PTB patients	211													2.4	LJ	Tertiary hospital
3	Rosha D & Kataria V K, 2001 [52]	Ranchi	1999–2000	Prev. treated PTB patients	667	24.1	2.3	4.2	2.1	1.5	3.2								LJ	Tertiary hospital (Armed forces)
4	Rai S P et al., 2007 [27]	Ranchi	2001–2004	Newly diagnosed PTB patients	769	18.0	0.4	1.4	1.7	0.4	3.0	1.6		1.6					LJ	Tertiary hospital (Armed forces)
5	Jain A et al. (a), 2008 [53]	Lucknow	2000–2002	Combined PTB patients	353	37.4	2.0	0.3	0.6	6.0		19.3	29.0	11.0					LJ	Tertiary hospital
6	Jain A et al. (b), 2008 [54]	Lucknow	2002–2004	Combined PTB patients	686	38.2	29.7	20.7	17.8	27.7		19.8	25.5	13.2					LJ	Primary, Secondary and Tertiary healthcare
7	Rawat J et al., 2009 [55]	Dehradun	2002–2006	Prev. treated PTB patients	180	62.8	5.00					57.2	57.2						LJ	Tertiary hospital
8	Datta B S et al., 2009 [56]	Kashmir	2003–2007	Newly diagnosed PTB patients	910							5.7						0.9	MGIT 960	Primary, Secondary and Tertiary healthcare
9	Mathuria J P et al. (1), 2013 [38]	Sawai Madhopur	2004–2007	Combined PTB patients	48	22.92	4.17		2.08			14.58	66.67	7.14					LJ	District TB center
	Mathuria J P et al. (2), 2013 [38]	Buxar	2004–2007	Combined PTB patients	24	54.17	0.00		4.17			37.50	43.75	25.00					LJ	District TB center
10	Sharma S K et al. (b), 2011 [18]	Delhi	2005–2008	Prev. treated PTB patients	196		0.00	1.53				20.41	20.41						LJ	Primary and tertiary healthcare
11	Hanif M et al., 2009 [17]	Delhi	2006	Prev. treated PTB patients	2880	52.01	4.69	47.12	3.72	13.99		47.12	47.12						LJ	TB referral center
12	Magee M J et al., 2012 [57]	Delhi	2006	Combined PTB patients	53	47.17	7.55	1.89	5.66	3.77		13.21							LJ, MGIT 960	HIV outpatient clinics (private)
13	Angrup A et al., 2011 [58]	Delhi	2006–2007	Combined PTB patients	67		1.5		3.0	11.9		10.5							LJ	Patients from DOTS center and private clinics
14	Sethi S et al., 2013 [59]	Chandigarh	2006–2010	Combined PTB patients	219	26.5	11.0		3.2	11.9		17.8	27.6	9.9					LJ, MGIT 960	Tertiary hospitals
15	Myneedu V P et al. (a), 2011 [60]	Delhi	2007–2009	Prev. treated PTB patients	223							8.1						20.2	LJ	Tertiary hospital
16	Mishra J K et al., 2009 [61]	Varanasi	2007–2009	Combined PTB patients	51							21.6	26.5	11.8					Not specified	Tertiary hospital
17	Porwal C et al., 2013 [28]	Delhi	2007–2010	Prev. treated PTB patients	609							79.3	79.3		8.7	67.9	32.1	3.0	MGIT 960	Tertiary hospital
18	Sharma S K et al. (c), 2011 [62]	Delhi	2008–2009	Newly diagnosed PTB patients	177		1.7					1.1		1.1					LJ	Primary healthcare
19	Yadav R et al., 2013 [63]	Chandigarh	2008–2010	Combined PTB patients	171	48.5	5.9	1.2	4.7	14.0		17.0	33.3	5.9					LJ	Tertiary hospital
20	Gupta A et al. (a), 2011 [64]	Varanasi	2008–2010	Combined PTB patients	288	57.6						35.8							LJ	Tertiary hospital
21	Sagar T et al., 2013 [65]	Delhi	2009–2010	Combined PTB patients	36	5.6	2.8					2.8							LJ	Tertiary hospital
22	Singh N et al., 2014 [66]	Delhi	2009–2012	Prev. treated PTB patients	353							67.71	67.71						Not specified	RNTCP district centers
23	Khanna A et al., 2010 [67]	Delhi	2010	Combined PTB patients	194							53.6						3.1	MGIT 960	Referral cases from diagnostic center
24	Maurya A K et al., 2013 [45]	Lucknow	2011–2012	Combined PTB patients	125	64.8						36.0							BacT/ALERT and Geno-Type® MTBDR plus assay	Tertiary hospitals
25	Myneedu V P et al. (b), 2015 [68]	Lucknow	2011–2012	New PTB patients	340	23.2	7.1	0.9				5.3		5.3				0.3	LJ	District DOTS center
26	Singhal R et al. (b), 2015 [69]	Delhi	2011–2012	Prev. treated PTB patients	2038	29.8	7.3	4.6				18.0	18.0						Geno-Type® MTBDR plus assay	National Reference laboratory
27	Jain A et al. (c), 2012 [29]	Lucknow	2012	Prev treated PTB patients	361	36.0	17.7	0.6	1.1	5.0		36.0	36.0		15.2	65.5	34.6	3.1	LJ	Tertiary care center
28	Jain A et al. (d), 2014 [16]	Lucknow, whole UP	2009–2012	Prev. treated PTB patients	2496	54.4	7.8	0.9	1.6	5.5		27.8	27.8						LJ	Tertiary care center
29	Kumar P et al., 2015 [20]	Punjab	2012–2013	Combined PTB patients	545	53.2	9.4	18.0				25.9							Gento-type MTB DRP plus assay	Tertiary care center
30	Gupta H et al., 2013 [70]	Lucknow	2010–2011	New PTB patients	169	21.3	18.3	4.7	10.6	10.1		4.7		4.7					LJ	DOTS center
31	Prajapati S et al., 2016 [71]	Delhi	2010–2011	New PTB patients	127	20.47	3.15	0.79	1.6	3.1		3.9		3.9					MGIT 960	AIMS, Children hospital
32	Gupta A et al. (b), 2015 [72]	Varanasi	2015	Combined PTB patients	354							29.4							LJ	Tertiary hospital
SOUTH INDIA																				
33	Vasanthakumari R & Jagannath K, 1997 [73]	Chennai	1997	Prev. treated PTB patients	162	63.0						20.4	20.4						LJ, DST, MIC	Tertiary hospital
34	Paramasivan C N et al. (a), 2000 [74]	Tamil Nadu	1997	Combined PTB patients	400	20.0	7.8	0.5	0.5	1.8		4.3	1.0	81.3					LJ	Reference laboratories across the state
35	Subhash H S et al., 2003 [75]	Vellore	1997–1999	Combined PTB patients	291	54.0													Not specified	Tertiary hospital
36	Deivanayagam C N et al., 2002 [76]	Chennai	1997–2000	Prev. treated PTB patients	618	80.1	66.3	55.5	46.4	35.6		54.9	54.9						LJ	Tertiary hospital
37	Vijay S et al., 2004 [40]	Bangalore	1999	Newly diagnosed PTB patients	271	27.7	3.7	0.4	0.4	13.3		2.2		2.2					LJ	District TB Centre
38	Paramasivan C N et al. (b), 2002 [77]	North Arcot; Raichur	1999	Combined PTB patients	587	27.8	6.1		0.2	3.2		6.3	81.5	2.7					LJ	District TB Centers
39	Ravindran C et al., 2006 [78]	North Kerala	1999–2000	Newly diagnosed PTB patients	45	17.8	4.4					8.9		8.9					LJ	Outpatient clinics
40	Velayutham B R et al., 2014 [79]	Tiruvallur	1999–2004	Combined PTB patients	2408	20.7	15.7	3.8		10.1		3.5	10.6	1.5					LJ	District TB Center
41	TRC_ICMR, 2001 [80]	Chennai	2001	Prev. treated PTB patients	1817	21.0	9.4	0.2		4.0		5.3	5.3						LJ	TB research center
42	Anuradha B et al., 2006 [81]	Hyderabad	2001–2003	Combined PTB patients	909	7.0	2.4	1.0	0.3	0.9		1.5	5.6	0.4					LJ	Tertiary hospital
43	Paramasivan C N et al. (c), 2010 [31]	Chennai, across India	2001–2004	Prev. treated PTB patients	2816	74.9	5.6	0.6	0.04	2.0		53.2	53.2		5.9	65.3	34.7	2.5	LJ	TB research center
44	Joseph M R et al., 2007 [39]	Eranakulam	2003	Newly diagnosed PTB patients	305	27.9	2.6	1.0		17.4		2.0		2.0					Not specified	Designated microscopy centers
45	James P et al., 2011 [82]	Vellore	2003–2007	Prev. treated PTB patients	177	72.9	5.7			2.8		58.2	58.2						Not specified	Tertiary hospital
46	Rajasekaran S et al., 2009 [32]	Chennai	2004–2007	Combined PTB patients	2927	56.4						33.9						1.6		Tertiary hospital
47	Nagaraja C et al., 2012 [83]	Bangalore	2005–2010	Combined PTB patients	309							72.5							LJ	Tertiary hospital
48	Therese K L et al. (a), 2012 [84]	Chennai	2007–2009	Combined PTB patients	9	55.6	11.1				22.2	22.2							BACT-EC	Tertiary hospital
49	Duraisamy K et al., 2014 [85]	Kerala	2009–2010	Prev. treated PTB patients	1207							14.8	14.8						LJ	Records from state RNTCP
50	Bhat S et al., 2010 [86]	Mangalore	2010	Newly diagnosed PTB patients	50	82.0	10.0	6.0	6.0	32.0		4.0		4.0					LJ	Tertiary hospital
51	Kandi S et al., 2013 [87]	Hyderabad	2010–2011	Prev. treated PTB patients	84	50.0	13.1	2.4		1.2		33.3	33.3						LJ	Tertiary hospital
52	Therese K L et al. (b), 2012 [88]	Chennai	2011	Combined PTB patients	166	45.2	2.4	0.6		3.6	12.1	6.6							BACTEC MicroMGIT	Tertiary hospital
53	Selvakumar N et al., 2015 [26]	Tamil Nadu	2011–2012	Combined PTB patients	1934	26.6	9.6	1.0				6.00	13.2	1.8	1.7	93.9	6.1	0.2	LJ	Designated microscopy centers
54	Gaude G S et al., 2014 [19]	Belgaum	2011–2012	Combined PTB patients	66	69.7		10.6		3.0		36.4							LJ	Tertiary hospital
55	Thirumurugan R et al., 2015 [89]	Puducherry	2011–2013	Combined PTB patients	127	70.9		16.5				54.3							LJ	Outpatient clinics
56	Udaykumar A J et al.,2014 [90]	Bangalore	2014	Combined PTB patients	61							32.8						14.8	LJ	Tertiary hospital
57	Ranganath R et al., 2013 [91]	Mysore	2011–2012	Prev. treated PTB patients	125	57.6	12.0	8.0	3.2	1.6		25.6	25.6						MB/BacT system	Tertiary hospital
EAST INDIA																				
58	Mahadev B et al., (1), 2005 [92]	Hoogli,WB	2000–2001	Newly diagnosed PTB patients	263	16.7	2.3			6.5		3.0		3.0					LJ	Designated microscopy centers
	Mahadev B et al. (2), 2005 [92]	Mayurbhanj, Orissa	2000–2001	Newly diagnosed PTB patients	282	5.3	1.1			2.9		0.7		0.7					LJ	Designated microscopy centers
59	Chakraborty N et al., 2010 [93]	Kolkata	2007–2008	Combined PTB patients	120	35.8	4.2	4.2	1.7	5.0		15.0						3.3	LJ	Tertiary hospital
60	Lahiri S et al., 2015 [94]	Kolkata	2011–2012	Combined PTB patients	917	96.3	1.3	4.7	0.2	0.8		80.8							LJ	State Intermediate reference laboratories
61	Singhal R et al. (a), 2014 [24]	North Eastern states	2012	Prev. treated PTB patients	339		8.6	8.3				53.4							Geno-Type® MTBDR*plus* assay	Designated microscopy centers
WEST INDIA																				
62	Chand K et al. (a), 2000 [95]	Pune	1995–1998	Combined PTB patients	1120	17.1	1.3	2.6	0.1	4.6	0.4	3.0							LJ	Tertiary hospital (Armed Forces)
63	Shah A R et al., 2002 [96]	Ahemdabad	2000–2001	Prev. treated PTB patients	822	58.6	7.5	1.0	0.5	1.5		9.3	9.3						LJ	DOTS center
64	Chand K et al. (b), 2006 [97]	Pune	2000–2003	Combined PTB patients	172	12.8	1.7	0.6	0.6	3.5	0.6	2.9							LJ	Tertiary hospital (Armed forces)
65	Pereira M et al., 2005 [98]	Pune	2000–2004	Newly diagnosed PTB patients	70	18.6	10.0		4.3	4.3		5.7		5.7					BACTEC MGIT 960	Tertiary hospital
66	Almeida D et al., 2003 [21]	Mumbai	2003	Combined PTB patients	300	48.7	3.0	1.3		5.7		26.7	48.0	11.4					LJ	Tertiary care center
67	Menon S et al., 2012 [42]	Mumbai	2005–2009	Combined PTB patients	673	85.9	2.4	5.9		5.8		47.6							DST not specified	Tertiary care center
68	D’souza D T et al., 2009 [23]	Mumbai	2004–2007	Combined PTB patients	724	70.4	9.7	1.0			0.1	29.3	41.1	23.7					Buddemeyer technique	Cases from district TB registers.
69	Ramachandran R et al., 2009 [99]	Gujarat	2005–2006	Combined PTB patients	2618	31.3	7.9	0.5	0.1	9.3		8.4	17.4	2.4				0.3	LJ	Designated microscopy centers
70	Dalal A et al., 2015 [100]	Mumbai	2005–2013	Prev. treated PTB patients	340	29.4						29.4	29.4						MGIT 960	Private hospitals
71	Pradhan N et al., 2013 [101]	Pune	2008–2010	Prev. treated PTB patients	249	53.4						53.4	53.4		10.0	52.0	48.0	4.8	LJ	Tertiary care center
72	Jain S K et al., 2013 [15]	Pune	2010–2012	Newly diagnosed PTB patients	3	66.7						33.3		33.3					LJ, MGIT-960	Tertiary care center
73	Vadwai V et al., 2011 [102]	Mumbai	2011	Combined PTB patients	250	77.6	4.0					73.6	77.2	68.3					MGIT	Tertiary care center
CENTRAL INDIA																				
74	Hemvani N et al., 2001 [30]	Indore	1987–1996	Combined PTB patients	1426	88.0						8.1							LJ	Tertiary care center
75	Bhat J et al. 2015 [25]	Gwalior, Shivpuri	2012–2013	Combined PTB patients	475	26.95	5.05	0.8	0.6	11.4		4.0	3.0	1.1					LJ	Vulnerable Tribal Group

*Abbreviations* **:** *DR-TB* Drug resistant tuberculosis, *DOTS* Directly Observed Treatment, Short Course, *DST* Drug susceptibility testing, *E* Ethambutol, *FQ* Fluoroquinolone, *H* Isoniazid, *Inj* Aminoglycoside injectable, *L-J* Löwenstein-Jensen method, *MDR-TB* Multidrug-resistant tuberculosis, *MGIT* Mycobacteria Growth Indicator Tube, *MTB Mycobacterium tuberculosis*, *PTB* Pulmonary tuberculosis, *R* Rifampicin, *S* Streptomycin, TRC, ICMR, Tuberculosis Research Centre, Indian Council of Medical Research, *XDR TB* Extensively drug-resistant tuberculosis, *Z* Pyrazinamide

^a^Total no. of drug resistant cases/ Total no. of MTB isolates

^b^Total no. of multidrug resistant cases/ Total no. of MTB isolates

^c^Total no. of multidrug resistant cases in previous treated cases/ Total no. of MTB isolates from previously treated patients

^d^Total no. of multidrug resistant cases in newly diagnosed cases/ Total no. of MTB isolates from newly diagnosed patients

^e^Total no. of Pre-XDR cases/ Total no. of MTB isolates

^f^Total no. of XDR cases/ Total no. of MTB isolates

Drug resistance (including DR-TB, MDR, pre-XDR and XDR) was reported by 26 studies for a total of 20,695 MTB isolates during the decade 1 and by 49 studies for 24,381 MTB isolates in the decade 2. Of these total isolates subjected to drug susceptibility testing (DST), 23,279 (51.6%) isolates were from previously treated patients and 11,401 (25.3%) from newly diagnosed cases (includes studies exclusively reporting previously treated and newly diagnosed isolate numbers and those reporting combined isolate numbers with a break-up by category). The remaining 10,396 (23.1%) were isolates from combined cases (wherein a break-up of isolate number from previously treated and new cases were not available).

**Fig. 2:**
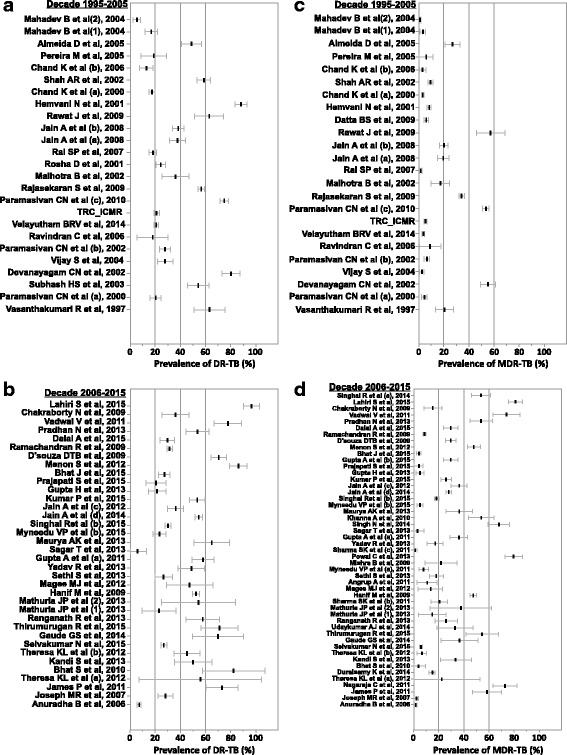
Forest plot of prevalence of DR-TB and MDR-TB. (**a**) Decade 1995–2005 (DR-TB) (**b**) Decade 2006–2015 (DR-TB) (**c**) Decade 1995–2005 (MDR-TB) (**d**) Decade 2006–2015 (MDR-TB). Abbreviations: DR-TB, drug resistant tuberculosis; MDR-TB, multidrug resistant tuberculosis

The Jain SK et al., 2015 study [15] from West India was considered as an outlier and excluded from analysis due to insufficient sample. The prevalence of DR-TB was found to be higher in the more recent study decade (decade 2), with 77.8% of published studies (28/36 studies) reporting a prevalence rate of more than 20%, as compared to 60.0% studies (15/25 studies) conducted during decade 1 (Fig. 2). This increasing trend in prevalence across the two decades was also noted for MDR-TB. Among studies conducted in decade 2, a prevalence of >20% was reported for 44.9% (22/49) studies versus 20.8% (5/24) studies in decade 1 (Fig. 2). Overall, of the 75 studies included in this analysis that tested 45,076 isolates for possible suspicion of resistance for various reasons, over 40% isolates were confirmed positive for resistance to any of the first-line anti-TB drugs.

### Subgroup analysis (decade and region-wise) for the prevalence of DR-TB and MDR-TB

**Fig. 3:**
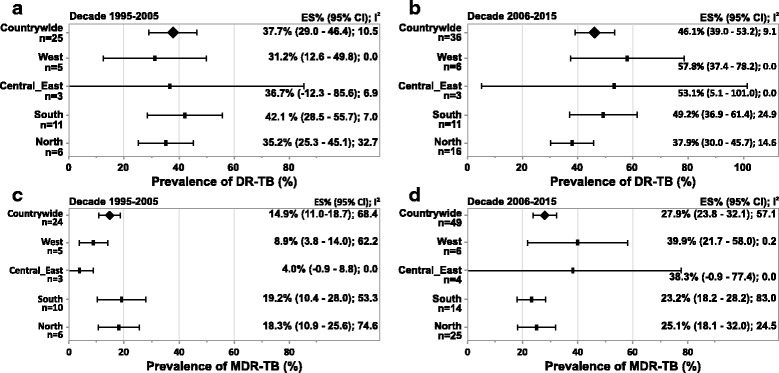
Subgroup analysis – prevalence of DR-TB and MDR-TB. (**a**) Decade 1995–2005 (Region-wise, DR-TB) (**b**) Decade 2006–2015 (Region-wise, DR-TB) (**c**) Decade 1995–2005 (Region-wise, MDR-TB) (**d**) Decade 2006–2015 (Region-wise, MDR-TB). Abbreviations: CI, confidence interval; DR-TB, drug resistant tuberculosis; ES, estimate; MDR-TB, multidrug resistant tuberculosis; n, number of studies. Notes: Negative I^2^ was set to zero. Any missing data means that studies conducted in that region did not present results eligible for inclusion in this analysis

The countrywide estimates for DR-TB was 37.7% (95% CI = 29.0; 46.4, *n* = 25) during decade 1, and a higher prevalence of 46.1% (95% CI = 39.0; 53.2, *n* = 36) was reported in decade 2. Overall, the prevalence estimate over the 20-year study period was 42.6% (95% CI = 37.2; 48.0, *n* = 61) (Table 2). The prevalence of DR-TB was highest in South India (42.1% [95% CI = 28.5; 55.7, *n* = 11]) and lowest in the Western region (31.2% [95% CI = 12.6; 49.8, *n* = 5]) during decade 1 (Fig. 3). In decade 2, West India (57.8% [95% CI = 37.4; 78.2, *n* = 6]) had the highest prevalence of DR-TB cases, and North India reported the lowest (37.9% [95% CI = 30.0; 45.7, *n* = 16]). The countrywide prevalence of MDR-TB also increased from the earlier decade (14.9% [95% CI = 11.0; 18.7, *n* = 24]) to decade 2 (27.9% [95% CI = 23.8; 32.1, *n* = 49]) and the prevalence for the 20-year period was 23.3% (95% CI = 20.5; 26.1, *n* = 73) (Table 2). MDR-TB, was most prevalent in the northern states (18.3% [95%CI = 10.9; 25.6, *n* = 6]) and least in the central and eastern states (4.0% [95% CI = −0.9; 8.8, *n* = 3]) during decade 1 (Fig. 3). Whereas, in decade 2, West India reported the highest number of cases for MDR-TB (39.9% [95% CI = 21.7; 58.0, n = 6]) and South India had the least (23.2% [95% CI = 18.2; 28.2, *n* = 14]).

**Table 2:** Status of drug-resistant tuberculosis in India

Drug resistance	n	Prevalence estimate (95% CI)	Heterogeneity test (I^2^)
1995 to 2015			
Any drug-resistance	61	42.6% (37.2; 48.0)	14.4
Multidrug resistance	73	23.3% (20.5; 26.1)	69.2
Previously treated	37	33.7% (27.9; 39.5)	29.0
Newly diagnosed	30	4.8% (3.7; 5.9)	79.3
Mono-drug resistance			
Isoniazid	53	7.2% (5.9; 8.4)	72.5
Streptomycin	40	6.7% (5.4; 8.0)	67.4
Rifampicin	42	4.6% (3.8; 5.5)	91.3
Ethambutol	31	1.6% (1.2; 2.0)	92.0
Decade 1: 1995 to 2005			
Any drug-resistance	25	37.7% (29.0; 46.4)	10.5
Multidrug resistance	24	14.9% (11.0; 18.7)	68.4
Previously treated	13	29.8% (20.7; 39.0)	45.0
Newly diagnosed	13	4.1% (2.7; 5.6)	70.2
Mono-drug resistance			
Isoniazid	21	8.6% (6.2; 10.9)	83.7
Streptomycin	18	6.7% (5.0; 8.5)	81.1
Rifampicin	15	3.6% (2.5; 4.7)	94.7
Ethambutol	13	1.9% (1.2; 2.6)	96.1
Decade 2: 2006 to 2015			
Any drug-resistance	36	46.1% (39.0; 53.2)	9.1
Multidrug resistance	49	27.9% (23.8; 32.1)	57.1
Previously treated	24	35.8% (29.2; 42.4)	36.3
Newly diagnosed	17	5.6% (3.8; 7.4)	82.1
Mono-drug resistance			
Streptomycin	22	6.8% (4.8; 8.8)	28.7
Isoniazid	32	6.2% (5.0; 7.5)	24.9
Rifampicin	27	5.1% (3.7; 6.6)	84.3
Ethambutol	18	1.7% (1.0; 2.3)	45.2

*CI* Confidence interval, *n* Number of studies

### Subgroup analysis (decade and region-wise) for the prevalence of MDR-TB among previously treated and newly diagnosed cases

Prevalence of MDR-TB was higher among previously treated patients than in newly diagnosed cases in both the decades. For the 20-year period, the countrywide estimates for MDR-TB was 33.7% (95% CI = 27.9; 39.5, *n* = 37) among the previously treated patients and 4.8% (95% CI = 3.7; 5.9, *n* = 30) among newly diagnosed cases (Table 2).

**Fig. 4:**
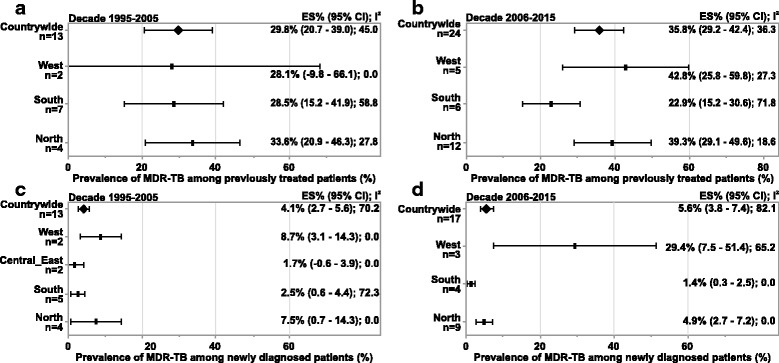
Subgroup analysis- prevalence of MDR-TB among previously treated and newly diagnosed patients. (**a**) Decade: 1995 to 2005 (previously treated patients) (**b**) Decade: 2006 to 2015 (previously treated patients) (**c**) Decade: 1995 to 2005 (newly diagnosed patients) (**d**) Decade: 2006 to 2015 (newly diagnosed patients). Abbreviations: CI, confidence interval; ES, estimate; MDR-TB, multidrug resistant tuberculosis; n, number of studies Notes: Negative I^2^ was set to zero. Any missing data means that studies conducted in that region did not present results eligible for inclusion in this analysis. Figure 4**b** and 4**d**: Countrywide prevalence includes 1 study from Central_East region (not presented individually)

The countrywide estimates for MDR-TB among previously treated patients was 29.8% (95% CI = 20.7; 39.0, *n* = 13) in decade 1 and 35.8% (95% CI = 29.2; 42.4, *n* = 24) in decade 2. MDR-TB in this population was highest in North India (33.6% [95% CI = 20.9; 46.3, *n* = 4]) and lowest in West India (28.1% [95% CI = −9.8; 66.1, *n* = 2]) in the earlier decade (Fig. 4). In decade 2, the western region (42.8% [95% CI = 25.8; 59.8, *n* = 5]) reported highest prevalence of MDR-TB among previously treated patients and southern region reported the lowest (22.9% [95% CI = 15.2; 30.6, *n* = 6]).

Among the newly diagnosed cases, the countrywide prevalence was 4.1% (95% CI = 2.7; 5.6, *n* = 13) during decade 1 and 5.6% (95% CI = 3.8; 7.4, *n* = 17) in decade 2. Highest estimate for MDR-TB was found in the West region (decade 1: 8.7% [95% CI = 3.1; 14.3, *n* = 2]; decade 2: 29.4% [95% CI = 7.5; 51.4, *n* = 3]) and lowest in the South (decade 1: 2.5% [95% CI = 0.6; 4.4, *n* = 5]; decade 2: 1.4% [95% CI = 0.3; 2.5, *n* = 4]) (Fig. 4).

### Prevalence of pre-XDR and XDR-TB

**Fig. 5:**
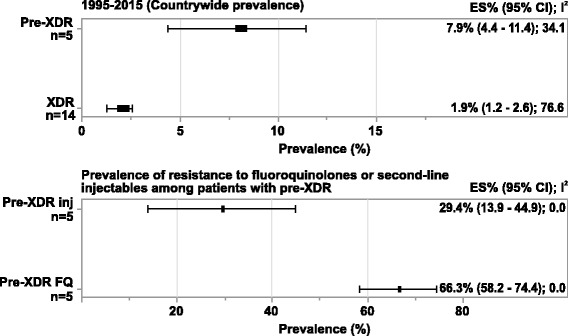
Subgroup analysis- Countrywide prevalence of Pre-XDR and XDR-TB. Abbreviations: CI, confidence interval; ES, estimate; FQ, Fluoroquinolone; Inj, aminoglycoside injectable; XDR-TB, extensively drug-resistant TB; n, number of studies. Notes: Negative I^2^ was set to zero. Any missing data means that studies conducted in that region did not present results eligible for inclusion in this analysis

The countrywide prevalence of pre-XDR TB over the 20-year period was 7.9% (95% CI = 4.4; 11.4, *n* = 5). A majority of these pre-XDR cases was due to resistance to fluoroquinolones (66.3% [95% CI = 58.2; 74.4, *n* = 5]). Prevalence of XDR-TB was notified in 14 studies and the countrywide prevalence was (1.9% [95% CI = 1.2; 2.6]) (Fig. 5). Due to limited data from published studies for pre-XDR and XDR-TB, a subgroup analysis stratified by regions and decades could not be performed.

### Subgroup analysis (decade and region-wise) for the prevalence of mono-drug resistance

The countrywide prevalence of mono-drug resistance revealed the highest rates for isoniazid across the 20-year period (7.2% [95% CI = 5.9; 8.4, *n* = 53) and during decade 1 (8.6% [95% CI = 6.2; 10.9, *n* = 21]). Resistance to streptomycin alone had the highest prevalence during decade 2 (6.8% [95% CI = 4.8; 8.8, *n* = 22]). Mono-drug resistance to ethambutol had the lowest prevalence over the 20-year timeframe (1.6% [95% CI = 1.2; 2.0, *n* = 31]), decade 1 (1.9% [95% CI = 1.2; 2.6, *n* = 13]) as well as decade 2 (1.7% [95% CI = 1.0; 2.3, *n* = 18)]) (Table 2). The country-wide estimates for rifampicin mono-drug resistance were 4.6% (95% CI = 3.8; 5.5, *n* = 42) over the 20-year period, 3.6% (95% CI = 2.5; 4.7, *n* = 15) in decade 1 and 5.1% (95% CI = 3.7; 6.6, *n* = 27) in decade 2 (Table 2).

**Fig. 6:**
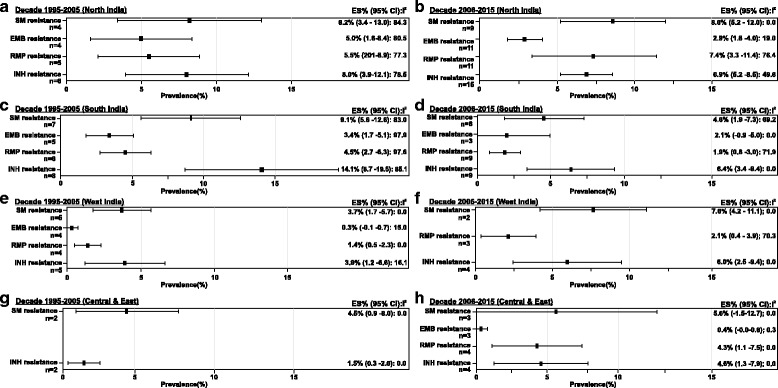
Subgroup analysis- prevalence of mono-drug resistance. (**a**) Decade 1995-2005 (North India) (**b**) Decade 2006-2015 (North India) (**c**) Decade 1995-2005 (South India) (**d**) Decade 2006-2015 (South India) (**e**) Decade 1995-2005 (West India) (**f**) Decade 2006-2015 (West India) (**g**) Decade 1995-2005 (Central & East India) (**h**) Decade 2006-2015 (Central & East India) Abbreviations: CI, confidence interval; EMB, ethambutol; ES, estimate; INH, isoniazid; MDR-TB, multidrug resistant tuberculosis; n, number of studies; RMP, rifampicin; SM, streptomycin. Notes: Negative I^2^ was set to zero. Any missing data means that studies conducted in that region did not present results eligible for inclusion in this analysis

Overall, the prevalence estimates for mono-drug resistance to streptomycin and isoniazid were generally high whereas, the prevalence of mono-drug resistance to ethambutol and rifampicin was low across all regions during both decades (Fig. 6).

## Discussion

This systematic review and meta-analysis attempted to demonstrate the geographical distribution of DR-, MDR- and XDR-TB and identify the high-risk regions and populations based on an analysis of published studies in India over the past two decades. To the best of our knowledge, the present study is the first to investigate the prevalence of DR-TB in India using systematic review of published studies. Pooled estimates for the countrywide prevalence of DR-TB and MDR-TB revealed a worsening trend between the two study decades. The estimates for MDR-TB subgroups from the present study were higher than the national estimates reported by the RNTCP for the year 2015 (15%, previously treated cases; 2.2%, newly diagnosed cases) and WHO estimates for India (16%, previously treated cases; 2.5%, newly diagnosed cases) [4, 7]. Estimates presented by global or national control programs are based on samples from government centers comprising of potentially susceptible populations or populations where the infection appearance or recurrence is monitored regularly and treated optimally. Therefore, estimates generated from an analysis of these samples may not be a true representation of the TB population in the real-world [16–18]. The present meta-analysis was based on results from published clinical studies conducted pan-India, reporting data for diverse patient populations at varied set-ups that include government tertiary care hospitals (not covered under RNTCP), outpatient clinics, private multispecialty hospitals and district level RNTCP centers. The data therefore, effectively entails regional influences and different epidemiological factors contributing to drug resistance and does not involve selective sampling of patients. However, it should be noted that the prevalence rates reported in the current analysis potentially reflect the status among suspected isolates referred for resistance testing and may not be reflective of prevalence rates of resistance in general, which may be lower.

Interrupted or irregular TB treatments are the strongest determinants for acquired mono-drug resistance and promote the risk of bacterial mutations that eventually culminate in relapses and MDR-TB [19, 20]. Regional analysis for estimates of drug resistance showed that the burden of DR- and MDR-TB in all regions (North, South, West, East and Central) increased over the 20-year period. West India had the lowest prevalence of DR-TB in decade 1 which increased considerably making it the region with the highest number of DR-TB cases in the 2006 to 2015 decade. The prevalence of MDR-TB in this region also increased between the two decades and the prevalence of primary MDR-TB in newly diagnosed smear-positive patients was higher in this region. The 12 studies from West of India included metropolitan cities such as Mumbai, Pune and major cities from Gujarat, highlighting the rapid emergence of DR- and MDR-TB in over-populated urban locales. Increased risk of infection transmission due to crowding, inadequacies in community TB control programs and most importantly, the high variability in the anti-TB treatment regimens prescribed by doctors, particularly in the private sector are some potential factors attributable to this upsurge [21, 22]. High rates of MDR-TB in Mumbai have previously been reported in individual studies involving RNTCP outpatients from municipal wards [23] and patients from a multispecialty private tertiary care hospital [21]. In contrast to the bigger cities in India, the studies in Central and East zones included population from rural and smaller towns. Among other factors, sparse population, access to free and supervised government aided medical centers and limited access to multiple doctors (leading to lesser variability in treatments) can be associated with the relatively lower prevalence of DR- and MDR-TB observed in this zone [21]. However, an overall underreporting of the DR- and MDR-TB burden due to difficult geographical terrain that limits accessibility to healthcare resources and poor socioeconomic status should not be overlooked [21, 24, 25].

Resistance to fluoroquinolones among pre-XDR-TB cases had the highest nationwide prevalence as compared with the rates for second-line aminoglycoside injectables. Easy access and indiscriminate use of fluoroquinolone antibiotics for other common non-TB infections are the most predictable risk factors for the development of resistance to these second-line drugs [26–31]. Findings from case studies suggest that short-term monotherapy with any fluoroquinolone can result in acquisition of resistance in MTB leading to serious implications that include poor MDR-TB treatment outcomes [32, 33]. Although, the estimates for XDR-TB over the 20-year period was low, of concern are the high rates of resistance to fluoroquinolones which have been regarded as one of the risk factors for the emergence of XDR-TB [28, 31, 34, 35]. India’s big share (63%) in the private TB market volume for second-line drugs is another major contributing factor for the high fluoroquinolone resistance observed [36]. Taking into account the minuscular share of the more preferred injectable second-line drugs (1% as opposed to 96% for fluoroquinolones [along with amoxicillin/clavunate]), fluoroquinolones are most likely to be used as monotherapy or even add-on to first-line anti-TB therapy instead of their recommended use as a second-line drug. Such irregularities in the usage of second-line drugs in private sector result in inadequate treatment for MDR-TB adversely impacting treatment outcomes and emergence of resistance [36, 37].

Mono-drug resistance to isoniazid and streptomycin were recorded at high levels and resistance to ethambutol alone had the least occurrence in India across both decades. Resistance to multiple first-line drugs underscores the importance of the implementation of the quadruple drug regimen for initial phase of tuberculosis treatment as advocated by DOTS [38]. The high levels of streptomycin resistance may be suggestive of its irrational use in non-DOTS treatment regimens at government and private set-ups [17, 39, 40]. Further, analysis of resistant strains have considered mono-drug resistance to isoniazid and streptomycin as factors that drive the development and amplification of additional resistance [41, 42].

Overall, these results emphasize on the importance of reinforcing DST in all patients previously exposed to anti-TB drugs to understand the drug resistance pattern and judiciously dispense standard or individualized chemotherapy for resistant cases. There is an impending need to curb the indiscriminate use of second-line drugs and advocate judicious use of newer drugs among physicians at various medical care set-ups to achieve better outcomes in patients with MDR-TB. The high prevalence of MDR-TB reported in the present study signifies the critical gaps in current treatment regimens and the need for fortification with better formulations comprising of newer drugs that have a distinct mode of action. In a country like India, where functioning of healthcare system heavily relies on the private sector, the adoption of newer drugs into government approved standardized regimens should be propagated unanimously and operational activities should be closely monitored for proper execution.

Some limitations of the present analysis should be considered. As the articles included for prevalence estimation did not encompass all states of India, these results may not truly represent the magnitude of DR-TB burden in India and should be interpreted with caution. In addition, the cumulative estimations of prevalence using a random-effect model may not completely invalidate the heterogeneity between studies. There was also a lack of adjustment for potential confounding factors such as socioeconomic status, age, gender etc. that could influence estimates derived from several studies. Further, it should be noted that an assessment of publication bias or selection bias was not performed.

Few noteworthy observations based on the review of published studies include the lack of standardized methods for DST adopted across India. The use of phenotypic and genotypic assays largely varied in public and private set-ups and was contingent on factors such as cost-effectiveness, availability of resources and sustaining infrastructure at various centers across India. This variability in turn introduces several incongruities such as, absence of standard definition of drug resistance and its different types and concerns pertaining to quality control, sensitivity, and reproducibility of results and validity of the laboratory techniques and could potentially affect the estimates from this meta-analysis [34, 43–46]. These observations emphasize the need to promote establishment and expansion of government endorsed laboratories with improved infrastructure that are capable of carrying out high quality, reliable and rapid turnaround DST.

Another grey area identified was the discordant recording of patient or clinical isolate data, which highlights the need for a standardized collection and reporting technique to aid better clinical correlations and decision making in India [47]. Some variables that contributed to these include differences in study durations and treatment strategies adopted across different regions and set-ups [46]. It is a challenge to understand the extent of nonadherence to medications or the quality of drugs taken by the patients since many were not on RNTCP recommended DOTS therapy [16]. The growing private healthcare sector in India is a major area of concern since these establishments involve the use and distribution of huge quantities of anti-TB drugs, with non-standardized treatment regimens that are not vigilantly supervised for adherence and completion [48]. These practices often lead to treatment interruptions and drug resistance is a consequence. In addition, timely notifications and efficient recording of patient details are regarded as early markers of community TB scenario and greatly support public healthcare programs. Inadequacies in these systems are therefore suggestive of looming danger [49]. In 2012, the Central TB Division (CTD) in collaboration with National Informatics Centre (NIC) initiated the implementation of a web-based application called ‘Nikshay’ [49]. This application primarily intends to create a robust database of all TB patients across India and enables access of this information to key policy makers, monitoring authorities and researchers who can positively impact treatment outcomes in TB-infected patients. The Government of India has mandated all private and government health establishments (outside the coverage of RNTCP) to ensure timely onward communication of patient details for the Nikshay repository [8].

There also exists a dire need for more regulated nationwide DRS based on standard epidemiological methods in India. Currently, sub-national DRS studies have been conducted in Gujarat, Maharashtra and South of India and the RNTCP is in the process of steering a nationwide initiative [8]. The RNTCP jointly with the National Tuberculosis Institute, Bangalore; U.S. Centers for Disease Control and Prevention (CDC) and WHO have constituted a nationwide survey comprising of representative populations of newly diagnosed and previously treated pulmonary TB cases. This initiative is expected to provide estimates that will be more generalizable to the entire nation and assist evaluations against global figures for improved understanding of the overall TB health situation in India.

## Conclusions

The pooled estimates from this study highlight the growing prevalence of DR- and MDR-TB in India that poses a new challenge to its clinical management and public health strategies. Future research involving assessment of clinical drug usage and identification of independent risk factors would be of great significance. Results from such studies along with robust prevalence estimates from the DRS may potentially help strengthen control measures, guide appropriate interventional and follow-up strategies in vulnerable populations and assist overall clinical decision-making.

## Abbreviations

CDC: Centers for Disease Control and Prevention
CI: Confidence interval
CTD: Central Tuberculosis Division
DOTS: Directly Observed Treatment, Short course
DR-TB: Drug resistant pulmonary tuberculosis
DST: Drug susceptibility testing
HIV: Human immunodeficiency virus
MDR: Multi-drug resistant
MTB: *Mycobacterium tuberculosis*
NIC: National Informatics Centre
pre-XDR: Pre-extensively drug-resistant
RNTCP: Revised National Tuberculosis Control Programme
RR-TB: Rifampicin-resistant tuberculosis
TB: Tuberculosis
WHO: World Health Organization
XDR: Extensively drug-resistant
